# A multidisciplinary approach to the treatment of children and adolescents with Beckwith-Wiedemann syndrome

**DOI:** 10.1192/j.eurpsy.2024.939

**Published:** 2024-08-27

**Authors:** M. Tripković, I. Bakija, D. Horvat, P. Lederer, I. Begovac

**Affiliations:** ^1^Department of Psychiatry and Psychological Medicine, University Hospital Centre Zagreb; ^2^Department for Integrative Psychiatry, Psychiatry Clinic Sveti Ivan, Zagreb, Croatia

## Abstract

**Introduction:**

Beckwith-Wiedemann syndrome (BWS) is a rare and complex congenital disorder characterized by a spectrum of symptoms and somatic findings. The prevalence of classic BWS is 1:26,000 births in Europe, and is equal in both sexes. The causes of the disorder are complex and are related to alterations in the expression of one or more genes in the region of chromosome 11. The heterogeneity of the clinical picture results in a spectrum of clinical features, the most common of which include: excessive growth of one side or certain parts of the body, macroglossia, abdominal wall deficits such as umbilical hernia, hypoglycemia, enlarged abdominal organs and an increased risk of developing certain types of tumors in adulthood.

**Objectives:**

The aim of this article is to highlight the importance of an early multidisciplinary approach in the management of children and adolescents with BWS.

**Methods:**

Using clinical practice and a review of the existing limited literature, we examined the complexity of the disease and the importance of psychiatric, psychotherapeutic, and psychological interventions in the treatment of children and adolescents with rare diseases such as BWS.

**Results:**

According to our clinical practice, a number of uncertain physical symptoms and possible complications may in some children with BWS lead to psychomotor retardation and lack of self-confidence due to the often impaired physical appearance. Affected children and adolescents are more likely to be exposed to abuse at school, show more behavioral and learning difficulties, difficulties in social adjustment, and resultant emotional difficulties. After initial genetic and pediatric treatment and subsequent regular monitoring, it is necessary to pay additional attention to the development of psychological sequelae in order to involve them and their families in psychotherapeutic treatment, and intervene in a timely manner so that they can achieve or maintain psychological stability and functionality. Many adolescent patients with BWS do not have significant somatic difficulties that would require pediatric intervention, but often present with symptoms of mental illness.

**Conclusions:**

Psychological stress in children and adolescents suffering from rare somatic diseases represents a negative experience of an emotional and social nature, which affects the course of the disease and interferes with the treatment. Due to a number of possible physical manifestations and outcomes of such diseases, extensive psychological support and care by child and adolescent psychiatrists and the entire medical team is required. A multidisciplinary approach is crucial in the treatment of these patients and results in improved functionality and quality of life.

**Disclosure of Interest:**

None Declared

